# Awake Prone Positioning in Moderate ARDS: A Case Report

**DOI:** 10.3390/clinpract16080151

**Published:** 2026-08-17

**Authors:** Kapilan Kalaruban, Aristomenis Exadaktylos, Vincent Ribordy, Mairi Ziaka

**Affiliations:** 1 Department of Internal Medicine, Thusis Hospital, 7430 Thusis, Switzerland; 2 Academic Department of Emergency Medicine, School of Medicine, University of Cyprus, Nicosia 1678, Cyprus; 3 Service des Urgences-SMUR, Fribourg Cantonal Hospital (HFR), 1708 Fribourg, Switzerland; vincent.ribordy@h-fr.ch; 4 Department of Emergency Medicine, Inselspital, University Hospital, University of Bern, 3010 Bern, Switzerland

**Keywords:** acute respiratory distress syndrome, awake prone position, community-acquired pneumonia, *Legionella pneumophila*, non-invasive ventilation

## Abstract

Background: *Legionella pneumophila* is a rare but severe cause of community-acquired pneumonia and can lead to acute respiratory distress syndrome (ARDS). Awake prone positioning (APP) has been recognized as an effective adjunct for non-intubated patients with hypoxemic respiratory failure, primarily studied in coronavirus disease 2019 (COVID-19) and other ARDS etiologies. Its application in *Legionella*-associated ARDS remains poorly documented. Therefore, in this work, we present a case of *Legionella*-associated ARDS successfully managed with APP, high-flow nasal cannula (HFNC), non-invasive ventilation (NIV), levofloxacin, and corticosteroids. Case presentation: A 62-year-old male with multiple comorbidities, including type 2 diabetes mellitus (T2DM) and a history of coronary artery bypass surgery, presented with a 3-day history of productive cough, exertional dyspnea, and general malaise. Oxygen saturation on admission was 90%, with fever and tachycardia. Inflammatory markers were markedly elevated. Chest computed tomography (CT) revealed extensive bilateral pulmonary infiltrates. Despite a negative urinary *Legionella* antigen test, sputum polymerase chain reaction (PCR) confirmed *Legionella pneumophila* on day 2. A Horowitz index of 147 mmHg on day 2 established moderate ARDS. The patient was treated with HFNC oxygen therapy, NIV, and APP for up to 12 h daily. Antibiotic therapy was initiated with amoxicillin/clavulanic acid and clarithromycin, subsequently streamlined to levofloxacin upon microbiological confirmation. Methylprednisolone 40 mg/day was administered for 8 days as adjunctive ARDS therapy. The patient demonstrated gradual clinical and respiratory improvement without requiring endotracheal intubation. Follow-up chest CT on day 7 showed regression of bilateral consolidations and ground-glass opacities (GGOs). The patient was transferred to pulmonary rehabilitation on day 11 and completed antibiotic therapy as an outpatient. Conclusions: This case illustrates the successful use of APP combined with HFNC and NIV alongside standard medical therapies, including appropriate antibiotics and corticosteroids, to avoid intubation in moderate ARDS secondary to *Legionella* pneumonia. Early initiation of APP may be a valuable strategy in *Legionella*-associated ARDS in carefully selected patients.

## 1. Introduction

*Legionella pneumophila* accounts for approximately 2–15% of hospitalized cases of severe community-acquired pneumonia and carries a mortality rate of up to 10% in severe disease, rising to 40–80% in immunocompromised patients [1,2,3]. The urinary *Legionella* antigen test (UAT), the most widely used rapid diagnostic test, detects only serogroup 1 strains with limited sensitivity in early or atypical presentations, potentially resulting in diagnostic delay. Sputum polymerase chain reaction (PCR) offers broader detection and may be critical when UAT is negative, but clinical suspicion remains high [4,5].

Legionellosis might cause intense pulmonary inflammation with diffuse alveolar damage, leading in a subset of patients to acute respiratory distress syndrome (ARDS), a life-threatening condition characterized by refractory hypoxemia, bilateral pulmonary infiltrates, and increased alveolocapillary permeability not related to cardiac failure or hypervolemia [6,7,8]. Recently, in a multicenter observational study, Dartevel et al. (2025) retrospectively investigated 162 patients with severe legionellosis and found that nearly 60% required invasive mechanical ventilation and approximately 50% fulfilled the criteria for a diagnosis of ARDS [9].

The Berlin definition classifies ARDS as mild, moderate, or severe based on the arterial oxygen partial pressure to inspired oxygen fraction ratio (PaO_2_/FiO_2_) ratio (Horowitz index), which guides therapeutic decisions [10]. Prone positioning has become standard evidence-based practice in mechanically ventilated patients with moderate-to-severe ARDS, demonstrated to significantly reduce 28-day mortality in the PROSEVA trial [11]. Physiologically, prone positioning reduces atelectasis by recruiting dependent dorsal lung regions, restoring aeration, eliminating pleural pressure gradients between nondependent and dependent regions, increasing functional residual capacity, reducing dead space, redistributing ventilation–perfusion (V/Q) relationships, and reducing intrapulmonary shunting [11,12,13,14,15,16]. More recently, due to the elevated demand for respiratory support during the coronavirus disease 2019 (COVID-19) pandemic, awake prone positioning (APP), performed in spontaneously breathing, non-intubated patients, has gained attention and emerged as a practical adjunct to high-flow nasal cannula (HFNC) and noninvasive ventilation (NIV) in acute hypoxemic respiratory failure, with studies demonstrating improved oxygenation and reduced intubation rates [11,17,18]. However, despite its first description in 1977, the application of APP in non-COVID-19 ARDS and acute hypoxemic respiratory failure, particularly in *Legionella*-associated ARDS, remains largely unreported [19,20].

We present a 62-year-old multimorbid patient with moderate ARDS secondary to *Legionella pneumophila* pneumonia, confirmed by sputum PCR despite a negative UAT, who was successfully managed with a combined non-invasive respiratory strategy, including APP, HFNC, and NIV, levofloxacin, and corticosteroids, thereby avoiding tracheal intubation.

## 2. Case Presentation

A 62-year-old male, married and working as a professional driver, was referred as an emergency by his general practitioner due to bilateral pneumonia on chest radiograph. He reported a 3-day history of productive cough, progressive dyspnea on exertion, markedly reduced general condition, and two episodes of vomiting. He denied fever, chest pain, or hemoptysis at home. His past medical history was significant for type 2 diabetes mellitus (T2DM; diagnosed in 2011, glycated hemoglobin A1c-HbA1c 7.6% on admission), coronary artery disease (3-vessel disease; status post 5-vessel coronary artery bypass graft in 2017 and multiple percutaneous coronary interventions), peripheral arterial occlusive disease, a known hymenoptera allergy (Grade III anaphylaxis on prior sting), and recurrent depressive episodes. He was a former heavy smoker (35 pack-years, cessation 2010) with a family history of cardiovascular disease and hypercholesterolemia. His regular medications included phenprocoumon, aspirin, insulin glargine, sitagliptin/metformin, empagliflozin, ezetimib/atorvastatin, esomeprazole, and escitalopram. The patient speaks minimal German, with Portuguese and Italian as his primary languages.

On examination, vital signs were as follows: blood pressure 131/84 mmHg, heart rate 111 beats per minute, body temperature 38.6 °C, and peripheral oxygen saturation (SpO_2_) 90% on room air. The patient was alert (Glasgow Coma Scale 15), oriented, and in reduced general condition. Pupils were isocoric and reactive. Pulmonary auscultation revealed vesicular breath sounds bilaterally with bilateral basal coarse crackles (right > left), without signs of obstruction or cyanosis. Cardiac examination demonstrated tachycardia, but regular and pure heart sounds. Abdominal examination revealed a soft, non-tender abdomen with normal bowel sounds. No peripheral edema or jugular venous distension was noted.

Laboratory evaluation on admission revealed markedly elevated inflammatory markers: C-reactive protein (CRP) 566.8 mg/L (reference range < 5 mg/L), leukocytosis at 11.5 G/L with a left shift (immature granulocytes 13.1%), and procalcitonin (PCT) 3.8 µg/L. Arterial blood gas (ABG) analysis demonstrated respiratory partial insufficiency with PaO_2_ 75 mmHg and arterial partial pressure of carbon dioxide (PaCO_2_) 30 mmHg on room air. Coagulation examination showed a supratherapeutic international normalized ratio (INR) of 3.4. Further abnormalities included mild hyponatremia (sodium 126 mmol/L), mildly elevated transaminases in keeping with hepatopathy in the context of systemic infection (aspartate aminotransferase (ASAT) 39 U/L, alanine aminotransferase (ALAT) 83 U/L, lactate dehydrogenase (LDH) 450 U/L, and mildly elevated troponin I at 22 ng/L with subsequent decreasing N-terminal pro-B-type natriuretic peptide (NT-proBNP) (985 ng/L to 343 ng/L), interpreted as demand-related rather than acute coronary syndrome. Renal function was preserved (creatinine 55 µmol/L, estimated glomerular filtration rate (eGFR) 107 mL/min/1.73 m^2^). Blood cultures obtained on admission showed no growth. Multiplex PCR for respiratory viruses (influenza A/B, respiratory syncytial virus, COVID-19) and *Mycoplasma pneumoniae* PCR were all negative. Urinary Legionella and pneumococcal antigens were both negative.

**Figure 1:**
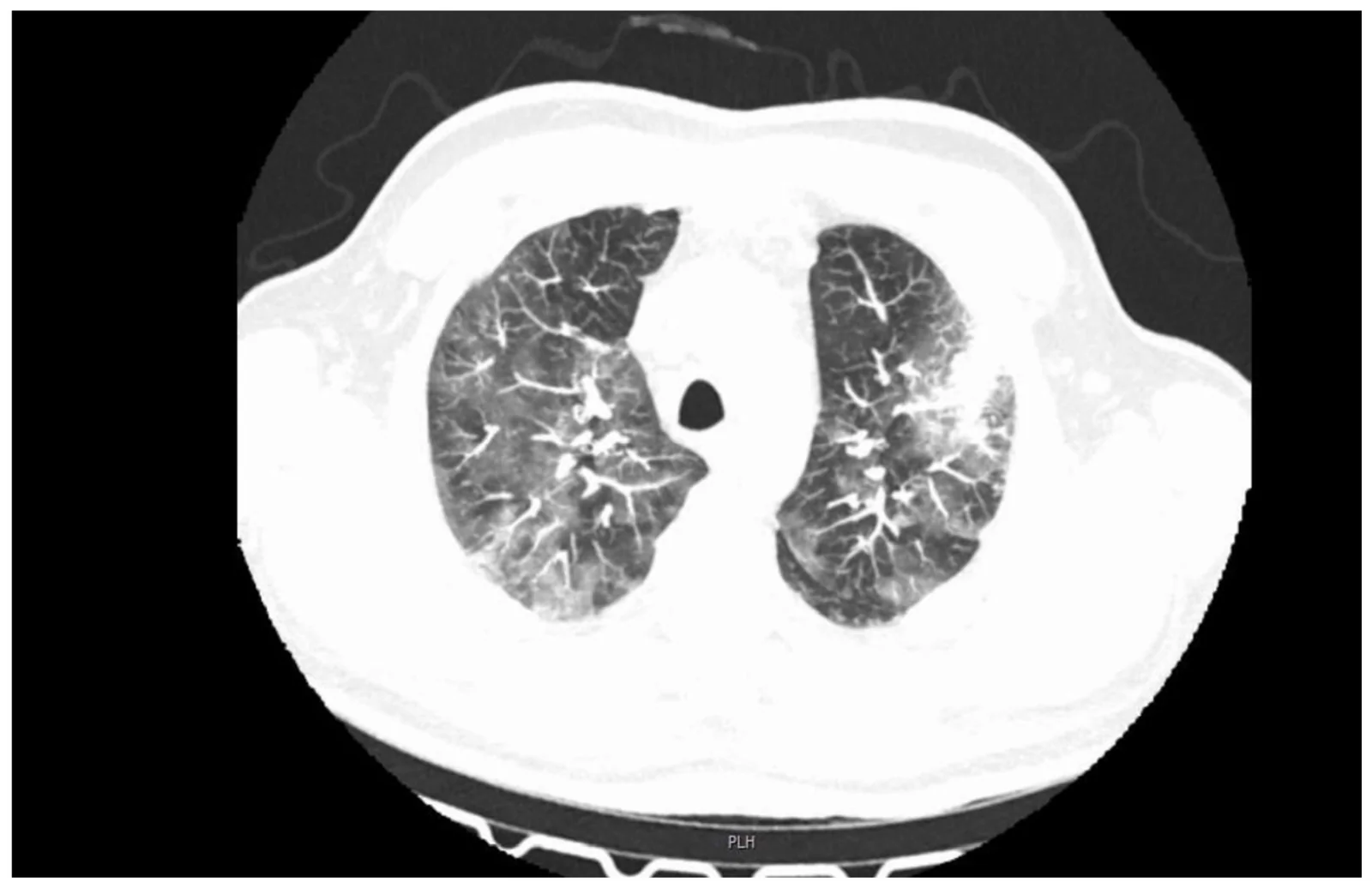
Extensive bilateral pulmonary infiltrates compatible with ARDS.

Chest CT performed on day 0 demonstrated extensive bilateral pulmonary infiltrates consistent with bipulmonary infiltrates suggestive of atypical pneumonia, with a differential diagnosis including ARDS and mediastinal lymphadenopathy (Figure 1). Empirical broad-spectrum antibiotic therapy was initiated with amoxicillin/clavulanic acid 2.2 g three times daily intravenously and clarithromycin 500 mg per os twice daily. On day 1, repeat ABG analysis yielded a Horowitz index (PaO_2_/FiO_2_) of 147 mmHg, confirming moderate ARDS according to the Berlin definition. HFNC oxygen therapy and NIV (Spontaneous-Pressure Support mode-SPN-PS, intermittently 3–4 times daily for 30–60 min per session) were initiated. Pressure support (PS) and positive end-expiratory pressure (PEEP) were adjusted to achieve a respiratory rate < 25 breaths/min, SpO_2_ > 92%, normocapnia, normal arterial pH, and optimal patient comfort. APP was commenced on hospital day 3 and progressively extended according to patient tolerance, reaching up to 12 h daily. The documented prolonged APP sessions were performed until hospital day 8. APP was well tolerated, without complications such as hemodynamic instability or respiratory deterioration. Methylprednisolone 40 mg/day intravenously was added as adjunctive ARDS therapy.

On day 2, sputum PCR returned positive for *Legionella pneumophila*, establishing the diagnosis of *Legionella* pneumonia despite the initially negative UAT. On day 4, antibiotic therapy was streamlined to targeted monotherapy with levofloxacin 500 mg twice daily intravenously for a planned 10-day course, continuing through day 14.

**Figure 2:**
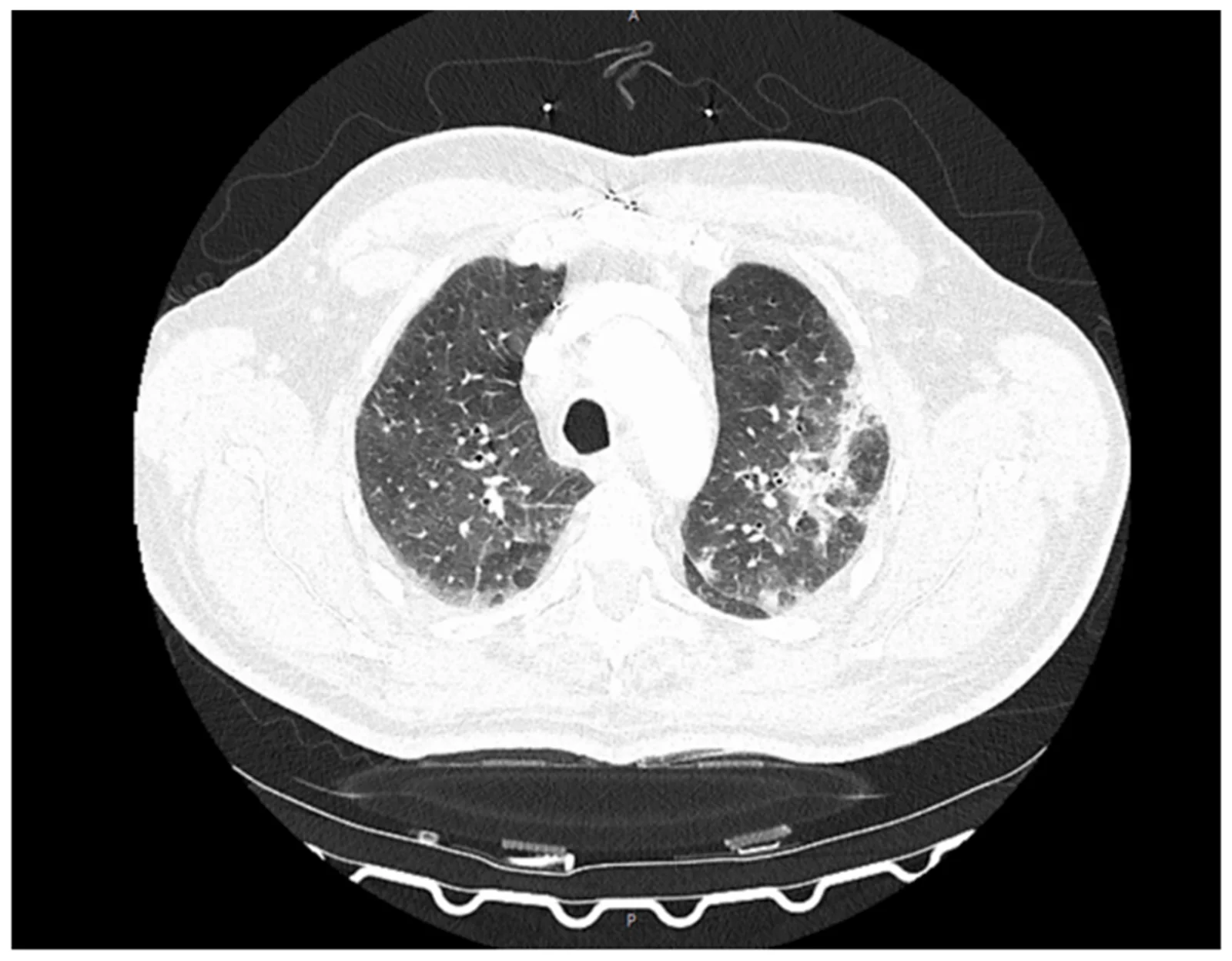
Serial chest CT scans demonstrating marked radiological improvement following combined therapeutic management with levofloxacin, systemic corticosteroids, HFNC, NIV, and awake APP.

Follow-up chest CT on day 7 demonstrated regression of bilateral consolidations and ground-glass opacities (GGOs) compared with admission, with residual parenchymal changes most prominent in the left upper lobe (Figure 2). New small bilateral pleural effusions were noted (right 22 mm, left 11 mm), alongside pre-existing mediastinal lymphadenopathy. The patient demonstrated gradual and sustained clinical improvement, with progressive mobilization from bed to chair and ambulation with supplemental oxygen. Methylprednisolone was discontinued on day 8 after completing the 8-day course. On day 11, the patient was transferred to a pulmonary rehabilitation facility for further convalescence on low-flow supplemental oxygen (2 L/min). Levofloxacin was continued orally until completion of the antibiotic course on day 13.

Table 1 summarizes the timeline of diagnosis, treatment, and key respiratory parameters throughout the hospitalization.

## 3. Discussion

This case demonstrates the successful management of moderate ARDS secondary to *Legionella pneumophila* pneumonia in a multimorbid patient using a multimodal non-invasive respiratory strategy, including HFNC, NIV, and APP, levofloxacin, and corticosteroids, thereby avoiding endotracheal intubation.

The pathophysiology of ARDS is complex and involves dysfunction of the alveolar-capillary barrier, dysregulated systemic and pulmonary inflammation, immune dysregulation, and responses to mechanical stimuli, all of which contribute to epithelial and alveolar injury following exposure to an inciting insult such as pneumonia, sepsis, or trauma [8,21].

It is strongly hypothesized that the development of ARDS or multiorgan dysfunction syndrome (MODS) in patients with legionellosis is multifactorial, with several contributing factors, including pathogen dissemination, the type IV Dot/Icm (T4SS; defective in organelle trafficking/intracellular multiplication) secretion system, bacterial virulence factors, and host immune responses, among others [22,23]. The Dot/Icm type IV secretion system, one of the major virulence factors of *Legionella pneumophila*, translocates more than 300 effector proteins into the cytoplasm of host cells. These effectors manipulate multiple host cellular processes, leading to the formation of a *Legionella*-containing vacuole (LCV) that supports intracellular bacterial replication [22,24,25,26,27]. In the lung, alveolar macrophages are the primary cellular targets of the T4SS effectors and provide a permissive intracellular niche for bacterial replication. T4SS effectors also target neutrophils, which can likewise harbor viable bacteria [22].

Accumulating evidence in recent years has highlighted that lung microbiota dysbiosis, defined as an imbalance between beneficial and harmful lung microbes, contributes to the pathophysiology of ARDS. In patients with ARDS, this dysbiosis is characterized by an increased bacterial burden, reduced microbial diversity, and alterations in microbiota composition [28,29,30]. In patients with severe legionellosis, the lung microbiome is altered, with an overgrowth of Firmicutes and Proteobacteria, a hallmark of lung dysbiosis, while low microbial diversity is associated with high bacterial biomass and enrichment of opportunistic bacterial and fungal pathogens, including *Pseudomonas*, *Stenotrophomonas*, and *Candida* [31,32]. These observations are consistent with recent research showing that patients with *Legionella* pneumonia may develop concomitant infections with pathogens such as *Staphylococcus aureus*, *Streptococcus pneumoniae*, *Pseudomonas* spp., Enterobacteriaceae, *Mycoplasma pneumoniae*, respiratory viruses, and *Haemophilus influenzae* [33,34]. Furthermore, experimental studies have demonstrated the presence of additional bacterial populations, including *Escherichia coli*, *Staphylococcus aureus*, and intestinal *Citrobacter* spp., in the respiratory tract of experimental models of *Legionella* pneumonia during the acute phase of disease [34].

Prone positioning has a well-established physiological rationale in ARDS. It redistributes gravitational pressure gradients within the lung, recruits previously atelectatic dorsal lung regions, reduces ventral over-distension, and promotes more homogeneous V/Q matching, ultimately decreasing intrapulmonary shunting and improving systemic oxygenation [16,35,36,37]. In the landmark PROSEVA trial, 16 h per day of prone positioning in mechanically ventilated patients with moderate to severe ARDS (Horowitz index < 150 mmHg) reduced 28-day mortality from 32.8% to 16.0% [11,38]. Nevertheless, these results should be interpreted with consideration of potential confounding factors. Specifically, cumulative fluid balance and vasoactive support were not assessed, and differences in the use of adjunctive therapies, including neuromuscular blockade, may have contributed to the observed outcomes [11].

In the COVID-19 era, APP in spontaneously breathing patients has emerged as an attractive adjunctive intervention in the management of non-intubated hypoxemic respiratory failure. A systematic review and meta-analysis by Fazzini et al. (2022), encompassing 1156 non-intubated patients, primarily with COVID-19-associated ARDS, demonstrated significant improvements in oxygenation indices and reduced mortality [17]. Recently, Harrois and colleagues (2025), in a randomized clinical trial, demonstrated a significant beneficial effect of APP on intubation rates and mortality in patients with COVID-19-associated acute hypoxemic respiratory failure [39]. However, the generalisability of existing data on the beneficial effects of APP in COVID-19-associated ARDS to non-COVID-19 ARDS should be interpreted with caution, as COVID-19-associated ARDS differs pathophysiologically from ARDS of non-COVID etiology, particularly in respiratory mechanics, including higher respiratory system compliance and increased dead space fraction [40,41].

The existing data on non-COVID-19 ARDS, particularly in the context of *Legionella* disease, are limited. Scaravilli et al. (2015) conducted a retrospective analysis of fifteen non-intubated patients with acute hypoxemic respiratory failure, showing improved oxygenation without significant alterations in respiratory rate or hemodynamics [42]. Ding et al. (2020) [19] showed, in a multicenter prospective cohort study, that early prone positioning in combination with HFNC or NIV was safe and effective in avoiding endotracheal intubation in 55% of patients with moderate-to-severe ARDS. However, all patients with a PaO_2_/FiO_2_ ratio < 100 mmHg while receiving NIV required endotracheal intubation, highlighting the severity of hypoxemia as a key determinant of prognosis. Across the four respiratory support strategies, improvement in oxygenation showed a progressive trend, with the efficacy ranking as follows: HFNC < HFNC + PP ≤ NIV < NIV + PP. Physiological studies in acute hypoxemic respiratory failure demonstrate that APP is associated with significant improvements in V/Q matching and reductions in shunt and dead space, and promotes a more homogeneous distribution of tidal volume (VT) toward dependent lung regions without influencing VT volume [43,44].

To the best of our knowledge, this represents the first reported case of *Legionella*-associated ARDS successfully managed with APP combined with HFNC and NIV. However, this observation should be interpreted in the context of disease severity. Ding et al. (2020) [19] reported a patient with *Legionella* pneumonia-associated ARDS treated with APP, HFNC, and NIV who ultimately required endotracheal intubation and invasive mechanical ventilation. Notably, that patient presented with severe ARDS (PaO_2_/FiO_2_ ratio of 73 mmHg), whereas our patient presented with moderate ARDS (PaO_2_/FiO_2_ ratio of 147 mmHg). These differences highlight the potential influence of ARDS severity on the response to APP-based strategies and support the need for careful patient selection and close monitoring. In our patient, APP was progressively increased from an initial duration of 2 h on day 2 to 12 h daily and was well tolerated throughout hospitalization, without adverse events such as pressure injuries, aspiration, or hemodynamic instability.

The use of corticosteroids, methylprednisolone 40 mg/day for 8 days, aligned with current evidence supporting adjunctive steroids in moderate-to-severe ARDS to attenuate pulmonary inflammatory injury [45,46]. In the context of *Legionella* pneumonia, steroids have been used as rescue therapy in severe disease, though their routine use remains debated given the risk of prolonged infection without adequate antibiotic cover [47]. However, recent guidelines recommend the use of corticosteroids in severe community-acquired pneumonia [45].

Although the existing evidence regarding the use of APP in severe hypoxemic community-acquired pneumonia remains limited and is based predominantly on observational studies with small sample sizes, case series, and case reports, its favorable safety profile, together with the recently published conditional recommendation from the Surviving Sepsis Campaign guidelines (2026) [48] suggests that APP may be considered as an adjunct to respiratory support in carefully selected patients with pneumonia-associated acute hypoxemic respiratory failure. In particular, patients with severe *Legionella pneumophila* pneumonia may represent an interesting population for future investigation. Given the marked hyperinflammatory cascade during the early phase of *Legionella* infection [49,50] early initiation of APP could theoretically improve lung recruitment, V/Q matching, and oxygenation, although this hypothesis has not yet been evaluated in clinical studies. Indeed, it has been well established that patients with the hyperinflammatory ARDS subphenotype may derive greater benefit from recruitment strategies [51]. Since prone positioning promotes more homogeneous lung inflation and may enhance alveolar recruitment [52], APP could theoretically have therapeutic potential in patients with severe *Legionella* pneumonia, particularly during the early phase of the disease. Furthermore, the radiological pattern of pneumonia may influence the response to APP. Patients with predominantly focal, dorsal-dependent consolidations may theoretically derive greater benefit from prone positioning than those with diffuse disease, although this hypothesis requires prospective validation [8,53,54]. Nevertheless, it should not be overlooked that alterations in transpulmonary pressure, defined as the difference between alveolar pressure and pleural pressure, represent the mechanical “stress” applied to the lungs and contribute significantly to patient self-inflicted lung injury (P-SILI). Therefore, intensive monitoring to identify patients with vigorous spontaneous breathing efforts, and consequently those at risk of exacerbating lung injury, is a crucial component of therapeutic management [16,19].

## 4. Limitations

Detailed physiological data assessing the immediate oxygenation response following each transition between respiratory support modalities (HFNC to NIV) and body positioning (supine to prone) are not available. Therefore, the individual contribution of NIV and APP to the observed improvement in oxygenation could not be determined. A limitation of this report is that, although APP sessions were documented, the exact duration of each individual session was not available. Therefore, the precise exposure to APP could not be fully quantified. Moreover, as a single case report, the generalizability of our observations is inherently limited. The concurrent use of multiple interventions, HFNC, NIV, APP, corticosteroids, and targeted antibiotic therapy, precludes attribution of the clinical benefit to any one modality. Individual patient factors, including the patient’s level of cooperation, cardiovascular reserve, and rapid antibiotic optimization, likely contributed to the favorable outcome. Prospective, controlled trials specifically investigating APP in *Legionella*-associated acute hypoxemic respiratory failure are warranted to validate these findings.

## 5. Conclusions

In conclusion, this case highlights two key clinical lessons: first, APP may be considered an adjunct to HFNC and NIV alongside standard medical therapies, including appropriate antibiotics and corticosteroids, in carefully selected patients with moderate ARDS secondary to *Legionella* pneumonia; however, its role in preventing intubation requires further investigation; and second, a negative urinary *Legionella* antigen test should not preclude further microbiological workup, particularly sputum PCR, in patients with severe community-acquired pneumonia and ARDS of uncertain etiology. Early initiation of APP, respiratory support, and timely antibiotic optimization based on definitive microbiological results appear to be key determinants of outcome in this challenging clinical scenario.

## Figures and Tables

**Table 1:** Clinical course, respiratory support, oxygenation parameters, and therapeutic interventions in severe *Legionella pneumophila* pneumonia complicated by ARDS.

Hospital Day	Clinical Course	Respiratory Support	Key Respiratory Parameters	PaO_2_/FiO_2_ (mmHg)	Therapeutic Measures
**0**	Emergency admission with bilateral pneumonia on CT and acute respiratory failure	Nasal cannula	Oxygen initiated and titrated up to 2 L/min	140	Amoxicillin/clavulanic acid 2.2 g i.v. three times daily; clarithromycin 500 mg i.v. twice daily
**1**	Moderate ARDS confirmed (Horowitz index 147 mmHg); awake prone positioning initiated	Escalation from nasal cannula to HFNC	HFNC 30 L/min, FiO_2_ 0.40	147	Methylprednisolone 40 mg/day i.v. initiated; insulin therapy introduced
**2**	Sputum PCR positive for *Legionella pneumophila*; urinary antigen negative	HFNC with NIV trial	HFNC 20–35 L/min, FiO_2_ 0.25–0.40; NIV trial (PS 2 cmH_2_O, PEEP 5–8 cmH_2_O, FiO_2_ 0.30)	147	Clarithromycin discontinued; targeted levofloxacin therapy initiated
**3**	Clinical improvement; targeted antibiotic therapy	Alternating NIV (SPN-PS), HFNC, and awake prone positioning	NIV PS 5–6 cmH_2_O, PEEP 8 cmH_2_O, FiO_2_ 0.30–0.40; HFNC 30 L/min, FiO_2_ 0.30	187 before APP; 221 after APP	Levofloxacin 500 mg i.v. twice daily continued
**7**	Follow-up CT: regression of consolidations and GGOs; small bilateral pleural effusions	Nocturnal NIV followed by nasal cannula	NIV PS 2 cmH_2_O, PEEP 8 cmH_2_O, FiO_2_ 0.30	224.6	Levofloxacin continued
**8**	Corticosteroid course completed; ongoing respiratory improvement	Nocturnal NIV → nasal cannula	NIV PS 3 cmH_2_O, PEEP 8 cmH_2_O, FiO_2_ 0.30; NC 2 L/min	372.3	Methylprednisolone discontinued
**11**	Transfer to pulmonary rehabilitation	Nasal cannula → room air	Oxygen discontinued after successful weaning	283	Oral levofloxacin 500 mg twice daily continued
**13**	Clinical recovery	Room air	—	Normal	Levofloxacin completed

ARDS, acute respiratory distress syndrome; APP, awake prone positioning; FiO_2_, fraction of inspired oxygen; GGOs, ground-glass opacities; HFNC, high-flow nasal cannula; NIV, non-invasive ventilation; PaO_2_/FiO_2_, arterial oxygen partial pressure to fraction of inspired oxygen ratio; PEEP, positive end-expiratory pressure; PS, pressure support.

## Data Availability

The original contributions presented in this study are included in the article. Further inquiries can be directed to the corresponding author.

## Abbreviations

ABG = arterial blood gas, ALAT = alanine aminotransferase, APP = awake prone position, ARDS = acute respiratory distress syndrome, ASAT = aspartate aminotransferase, BiPAP = bilevel positive airway pressure, CABG = coronary artery bypass graft, COVID-19 = coronavirus disease 2019, CPAP = continuous positive airway pressure, CRP = C-reactive protein, CT = computed tomography, eGFR = estimated glomerular filtration rate, FiO_2_ = fraction of inspired oxygen, GGO = ground-glass opacity, HbA1c = glycated hemoglobin A1c, HFNC = high-flow nasal cannula, i.v. = intravenous, LDH = lactate dehydrogenase, NIV = non-invasive ventilation, NT-proBNP = N-terminal pro-B-type natriuretic peptide, OI = oxygenation index (PaO_2_/FiO_2_), ORCID = Open Researcher and Contributor ID, p.o. = per os (oral), pCO_2_ = partial pressure of carbon dioxide, PCR = polymerase chain reaction, PCT = procalcitonin, pO_2_ = partial pressure of oxygen, RSV = respiratory syncytial virus, SARS-CoV-2 = severe acute respiratory syndrome coronavirus 2, SpO_2_ = peripheral oxygen saturation, T2DM = type 2 diabetes mellitus, UAT = urinary antigen test, V/Q = ventilation–perfusion, VT = tidal volume.
